# QTL Mapping Low-Temperature Germination Ability in the Maize IBM Syn10 DH Population

**DOI:** 10.3390/plants11020214

**Published:** 2022-01-14

**Authors:** Qinghui Han, Qingxiang Zhu, Yao Shen, Michael Lee, Thomas Lübberstedt, Guangwu Zhao

**Affiliations:** 1 The Key Laboratory for Quality Improvement of Agricultural Products of Zhejiang Province, College of Advanced Agricultural Science, Zhejiang Agriculture and Forestry University, Hangzhou 311300, China; qinghuihan@zafu.edu.cn (Q.H.); zqx@stu.zafu.edu.cn (Q.Z.); 2018801632020@stu.zafu.edu.cn (Y.S.); 2 Department of Agronomy, Iowa State University, Ames, IA 50011, USA; mlee@iastate.edu (M.L.); thomasl@iastate.edu (T.L.)

**Keywords:** low-temperature germination ability, IBM Syn10 DH population, QTL, maize, seed germination

## Abstract

Chilling injury poses a serious threat to seed emergence of spring-sowing maize in China, which has become one of the main climatic limiting factors affecting maize production in China. It is of great significance to mine the key genes controlling low-temperature tolerance during seed germination and study their functions for breeding new maize varieties with strong low-temperature tolerance during germination. In this study, 176 lines of the intermated B73 × Mo17 (IBM) Syn10 doubled haploid (DH) population, which comprised 6618 bin markers, were used for QTL analysis of low-temperature germination ability. The results showed significant differences in germination related traits under optimum-temperature condition (25 °C) and low-temperature condition (10 °C) between two parental lines. In total, 13 QTLs were detected on all chromosomes, except for chromosome 5, 7, 10. Among them, seven QTLs formed five QTL clusters on chromosomes 1, 2, 3, 4, and 9 under the low-temperature condition, which suggested that there may be some genes regulating multiple germination traits at the same time. A total of 39 candidate genes were extracted from five QTL clusters based on the maize GDB under the low-temperature condition. To further screen candidate genes controlling low-temperature germination, RNA-Seq, in which RNA was extracted from the germination seeds of B73 and Mo17 at 10 °C, was conducted, and three B73 upregulated genes and five Mo17 upregulated genes were found by combined analysis of RNA-Seq and QTL located genes. Additionally, the variations of *Zm00001d027976* (GLABRA2), *Zm00001d007311* (bHLH transcription factor), and *Zm00001d053703* (bZIP transcription factor) were found by comparison of amino sequence between B73 and Mo17. This study will provide a theoretical basis for marker-assisted breeding and lay a foundation for further revealing molecular mechanism of low-temperature germination tolerance in maize.

## 1. Introduction

As an important grain, forage and industrial raw material, maize planting area ranks first in worldwide. The germination and growth of maize, which was originated from tropical and subtropical areas, were seriously threatened by chilling injury in early spring. Under low temperature, germination rate was decreased in maize, while the elongation of plumule length, seedling length, and root length were inhibited [1]. The low seed vigor and poor low-temperature tolerance of cultivated varieties had led to irregular emergence and seedling absent in early spring. In order to cope with low temperatures, mulch film was used to avoid the injury of low temperatures in production, but which had the shortcoming of high cost and residue contamination. Therefore, cultivation of new maize varieties with strong low-temperature germination ability will be the fundamental way to improve maize germination rate in early spring [2]. However, the breeding progress of maize was hindered, because the germplasm or genes related to low-temperature tolerance had not been fully excavated and utilized during germination. 

In crops, the studies related to low-temperature germination tolerance are mainly focused on rice. Different rice populations have been used for quantitative trait locus analysis for traits of low-temperature germination tolerance, and there have been more than 30 QTLs mapped in past decades [3,4,5]. Furthermore, 53 QTLs were obtained in rice by GWAS, of which 20 QTLs overlapped with previous studies [6]. Additionally, the group found that *ossap16*, which is located at the GWAS-associated region, positively regulated the low-temperature germination tolerance of rice [6].

With the development of molecular marker technology, many QTLs controlling maize growth under different stress environments have been identified by linkage map in maize. Genetic studies showed that cold tolerance of maize was a quantitative trait controlled by micro effect polygenes and was easily affected by environmental conditions [7]. At seedling stage, several QTLs and candidate genes, regulating chilling tolerance, were excavated in maize. By F2:3 population of maize ETH-DH7 × ETH-DL3, a main QTL, in which the candidate gene *Agp2* was included, controlling dry weight of aboveground part and dark response rate was found on chromosome 6 under low temperature [8]. Under low temperature, 20 QTLs for shoot traits and 40 QTLs for root traits were located at maize seedlings by F2:3 population, which was constructed by Lo964 and Lo1016 parent lines [9]. Two QTLs, *bins3.01* and *bins6.03*, controlling leaf color were mapped by recombinant inbred lines (RILs), which were constructed by B73 and Mo17, under low-temperature [2]. By multi-parent advanced generation intercross (MAGIC) population, 858 SNPs, associated with the number of days from sowing to emergence, chlorophyll content and maximum quantum efficiency of photosystem II under cold conditions were mapped [10]. By genome-wide association analyses (GWAS), 275 significant loci were associated with the traits, such as number of days from sowing to emergence, relative leaf chlorophyll content, and quantum efficiency of photosystem II, using two panels of 306 dent and 292 European flint maize inbred lines [11]. In another study, two populations of RILs involving sweet corn inbred lines developed from B73 × P39 and B73 × IL14 h were used for mapping under low temperature, and 27 QTLs were linked to increment of days to emergence and F_0_, decrease in dry weight and Fv/Fm in RILs populations of B73 × P39 and 24 QTLs in the RILs populations of B73 × IL14 h [12]. Recently, 159 quantitative trait loci (QTL) for emergence and traits related to early growth were associated by GWAS in 836 maize inbreds under cold treatment [13]. In maize seedlings, the function of ICE1–CBF transcriptional cascade on cold stress had been widely studied in previous papers [14]. Different from excessive reports at the maize seedling stage, there were few associated analyses related to low-temperature germination ability. Combining 43,943 SNPs, 15 significant SNPs, including three overlapped loci, were associated with germination rate, root length, and shoot length of 300 inbred lines by GWAS under cold stress [1]. In our previous study, 15 QTLs, located on chromosomes 4, 5, 6, 7, and 9, regulating low-temperature germination ability were mapped by 243 Syn4 recombinant inbred line (RIL) populations, which were derived from B73 and Mo17 [15]. A number of QTLs related to cold tolerance were mapped by QTL or GWAS using different populations, but few candidate genes were studied in maize germination under low temperature. Fourteen traits of 222 diverse inbred lines were used for GWAS analysis, and 30 SNPs, which directly related to fourteen candidate genes, linked to low-temperature tolerance were detected. Joint analysis with RNA-Seq data, *Zm00001d039219* (putative MAPK encode genes) and *Zm00001d034319* (possible involving fatty acid metabolic process) may be responsible for maize germination under low temperature [16]. During seed germination, *ZmANN33* and *ZmANN35*, two annexin genes in maize were found positively regulated plasma membrane recovery under chilling stress [17]. Marker-assisted selection (MAS) makes it possible to improve the low-temperature tolerance of maize at germination stage [18]. In order to further study the QTLs controlling the low-temperature tolerance of maize, a high-density genetic linkage map comprised 6618 bin markers based on parents B73 and Mo17 was constructed, and 176 Syn10 DH lines were used as the mapping population to locate the QTLs in the study. For further screening key genes, RNA-Seq, which was conducted in B73 or Mo17 during germination at low-temperature, was used for joint analysis with QTL located genes. This study will provide a basis for molecular marker assisted breeding and functional study in low-temperature germination ability of maize.

## 2. Results

### 2.1. Phenotypic Analysis of Germination-Related Traits under Optimum-Temperature Condition and Low-Temperature Condition

**Figure 1:**
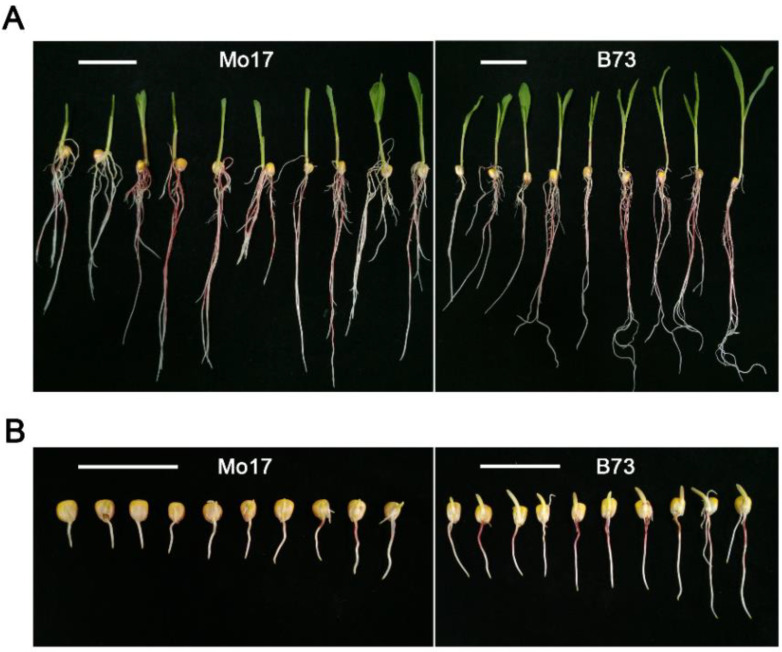
Morphological observation of Mo17 or B73 germinated seeds under optimum temperature or low temperature. (**A**) Photographs of Mo17 or B73 seeds/seedlings after imbibition for 7 days under optimum temperature (25 °C); (**B**) photographs of Mo17 or B73 germinated seeds after imbibition for 21 days under low temperature (10 °C). White scale bar represents 5 cm.

Eight traits, including the germination rate (GR), root length (RL), plumule length (PL), seedling length (SL), germination index (GI), vigor index (VI), simple vigor index (SVI), and average germination days (AGD), were measured under optimum (25 °C) and low (10 °C) temperatures (Table 1). The data showed that the value of each trait of B73 and Mo17 was significantly different at 10 °C (Figure 1), which suggested that they were suitable as parent materials for mapping the QTL related to low-temperature germination ability. Compared with Mo17, the average values of all traits of IBM Syn10 DH population were closer to B73 at the low temperature (Table 1).

**Figure 2:**
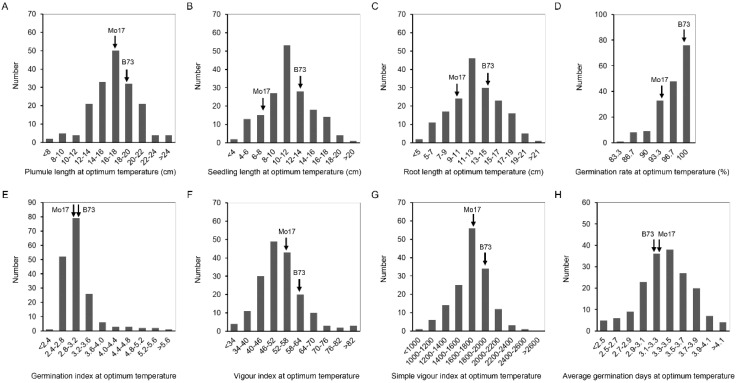
The histogram of frequency distribution of germination-related traits under 25 °C in IBM Syn10 DH population. (**A**) Plumule length at optimum temperature (OTPL); (**B**) seedling length at optimum temperature (OTSL); (**C**) root length at optimum temperature (OTRL); (**D**) germination rate at optimum temperature (OTGR); (**E**) germination index at optimum temperature (OTGI); (**F**) vigor index at optimum temperature (OTVI); (**G**) simple vigor index at optimum temperature (OTSVI); (**H**) average germination days at optimum temperature (OTAGD).

**Figure 3:**
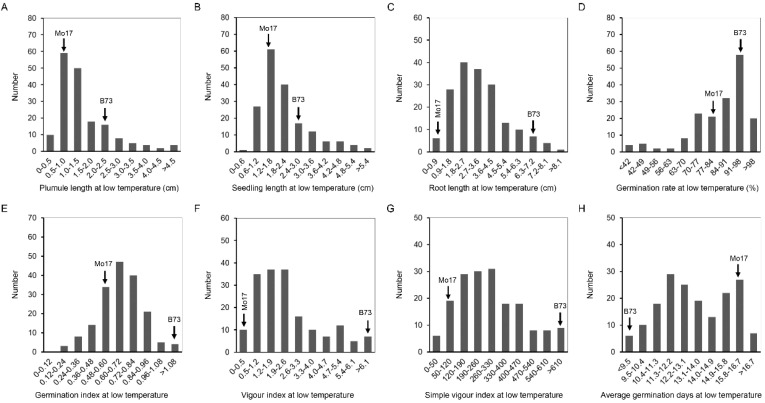
The histogram of frequency distribution of germination-related traits under 4 °C in IBM Syn10 DH population. (**A**) Plumule length at low temperature (LTPL); (**B**) seedling length at low temperature (LTSL); (**C**) root length at low temperature (LTRL); (**D**) germination rate at low temperature (LTGR); (**E**) germination index at low temperature (LTGI); (**F**) vigor index at low temperature (LTVI); (**G**) simple vigor index at low temperature (LTSVI); (**H**) average germination days at low temperature (LTAGD).

At 25 °C, the coefficient of variation (CV) of IBM Syn10 DH population was significantly different, in which the germination rate was the smallest (4%), and the seedling length was the largest (31.5%) (Table 1). Compared to the optimum-temperature condition, the CVs were larger at the low temperature, ranging from 17.1% (LTGR) to 94.2% (LTVI) (Table 1). IBM Syn10 DH population, constructed by B73 and Mo17, was highly diverse, which would provide abundant materials for breeding new maize varieties with low-temperature tolerance. The frequency of OTPL, OTSL, OTRL, and OTAGD formed a normal distribution (Figure 2), while the skewed distributions was presented in the frequency distribution histograms of OTGR, OTGI, OTVI, OTSVI, LTPL, LTSL, LTRL, LTGR, LTGI, LTVI, LTSVI, and LTAGD (Figure 2 and Figure 3). All these mean that low-temperature tolerance was a quantitative trait controlled by multiple genes.

Under optimum temperature, average germination days value was not correlated with plumule length, seedling length, root length, or germination rate. There was also no significant difference between plumule length and germination index. Under low temperature, LTPL, LTSL, LTRL, LTGR, LTGI, LTVI, LTSVI, and LTAGD were significantly different from each other at the level of *p* < 0.01 by Pearson correlation analysis (Table 2). Among these germination traits, LTAGD was negatively correlated with other traits.

### 2.2. Construction of a Molecular Linkage Map and QTL Analysis for Germination-Related Traits

**Figure 4:**
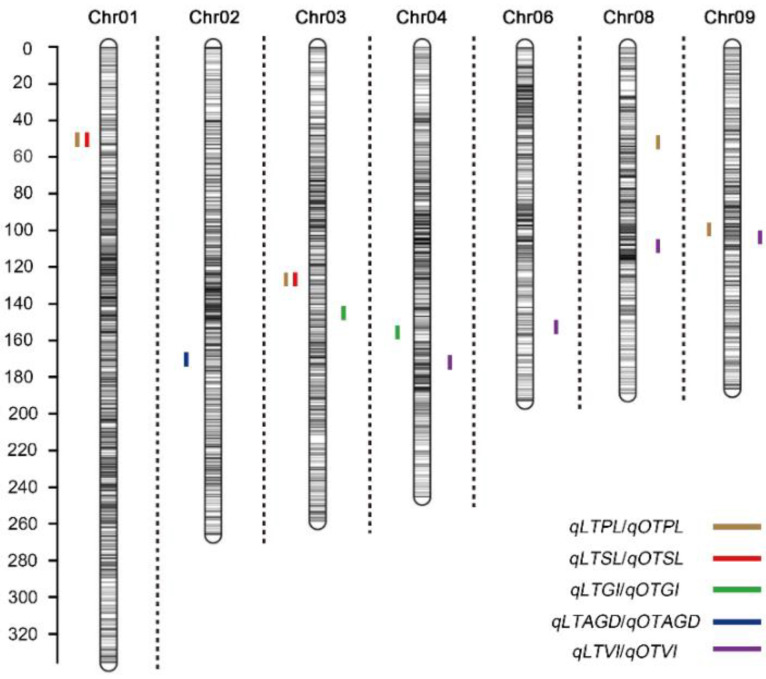
Chromosomal location of quantitative trait loci (QTL) for germination ability under low temperature (present in each chromosome left) and optimum temperature (present in each chromosome left) conditions in IBM Syn10 population. LTPL, LTSL, LTRL, LTGR, LTGI, LTVI, LTSVI, and LTAGD represent plumule length at low temperature, seedling length at low temperature, root length at low temperature, germination rate at low temperature, germination index at low temperature, vigor index at low temperature, simple vigor index at low temperature, and average germination days at low temperature, respectively; OTPL, OTSL, OTRL, OTGR, OTGI, OTVI, OTSVI, and OTAGD represent plumule length at optimum temperature, seedling length at optimum temperature, root length at optimum temperature, germination rate at optimum temperature, germination index at optimum temperature, vigor index at optimum temperature, simple vigor index at optimum temperature, and average germination days at optimum temperature, respectively.

A total of 6618 SNP markers were used to construct genetic linkage map. There were 1103, 780, 722, 763, 768, 567, 559, 521, 463, and 372 markers on 10 chromosomes, respectively [19]. The total length of linkage map of IBM Syn10 DH population was 2051.29 Mb, and the average physical distance was 0.31 Mb. At optimum temperature, six QTLs were located on chromosomes 3, 4, 6, 8, and 9 (Figure 4; Table 3). The LOD thresholds ranged from 2.51 to 4.53, which could explain 4.81~8.32% of the phenotypic difference. There were 3, 2, 1, and 1 QTLs controlling OTPL, OTSL, OTGI, and OTAGD, respectively. Under the low-temperature condition, LTPL contained three QTLs and explained 5.56~11.81% of the total phenotypic variation. *qLTSL1-1* had the largest LOD value of 8.17, additive effects of 0.47, and PVE value of 11.28% (Table 3). *qLTSL3-1* had a LOD value of 3.02 and additive effects of 0.27, which could explain 3.73% of the total phenotypic variation. *qLTGI4-1* had the lowest LOD value of 2.92, additive effects of −0.07 and PVE value of 6.82% (Table 3). All the values of additive effects of *qLTPLs* and *qLTSLs* were positive, suggesting that the alleles from B73 background promote seed germination. Average germination days were negatively correlated with other germination traits at 10 °C. *qLTAGD2*-1 had −0.64 of additive effect, suggesting the alleles from B73 background reduce average germination days and promote seed germination at low-temperature condition. It was interesting to note that there were two overlapping QTL regions. *qLTPL1-1* and *qLTSL1-1* overlapped between 18.575 and 18.875 Mb on chromosome 1. *qLTPL3-1* and *qLTSL3-1* overlapped between 187.475 and 187.725 Mb on chromosome 3 (Figure 4; Table 3).

### 2.3. Candidate Gene Screening

**Figure 5:**
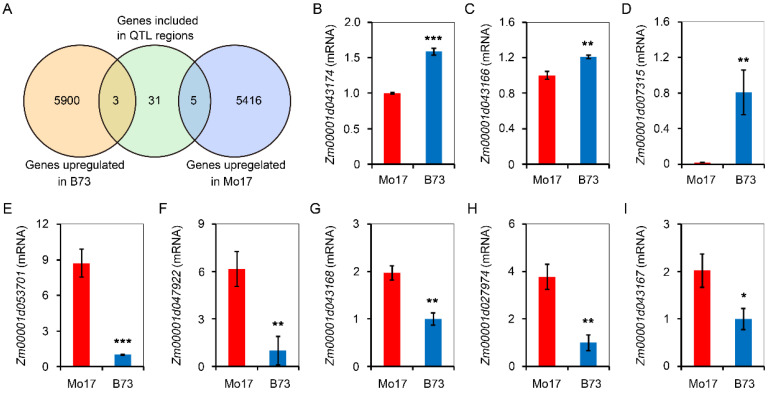
Comparison of QTL located genes and the genes upregulated in B73 or Mo17 seeds/seedling under low temperature. (**A**) Venn diagram showing overlapping genes between included by QTL region and upregulated in B73 or Mo17 seed/seedling. The seed/seedling used for RNA-Seq were extracted from B73 or Mo17 which was treated at 10 °C for 10 d. There are three replicates for each treatment. (**B**–**I**) Comparison of mRNA accumulation of *Zm00001d043174*, *Zm00001d043166*, *Zm00001d007315*, *Zm00001d053701*, *Zm00001d047922*, *Zm00001d043168*, *Zm00001d027974*, and *Zm00001d043167* in B73 or Mo17 seeds/seedling which was treated at 10 °C for 10 d. Values are means ± SE (n = 3). Asterisk represents significant difference. * *p* < 0.05, ** *p* < 0.01, *** *p* < 0.001 (Student’s *t* test).

A total of 39 candidate genes were included by five QTL clusters (Table S1), which overlapped by different low temperature traits. In order to mine genes controlling low-temperature germination, B73 and Mo17 seeds/seedlings, which were geminated at 10 °C for 10 d, were sent for RNA-Seq analysis. There were 5903 genes that were upregulated in B73, while 5421 genes were upregulated in Mo17. Combining analysis of QTL located genes and RNA-Seq data, there were three genes that were overlapped in B73 upregulated genes and QTL-located genes (Figure 5A). Additionally, there were five genes that were overlapped in Mo17-upregulated genes and QTL-located genes (Figure 5B). In further analysis, the mRNA levels of *Zm00001d043174*, *Zm00001d043166*, and *Zm00001d007315* were upregulated for 1.58, 1.21, and eight folds in B73 seeds/seedlings after germination for 10 days (Figure 5C–E), while *Zm00001d053701*, *Zm00001d047922*, *Zm00001d043168*, *Zm00001d043167*, and *Zm00001d027974* were upregulated for 8.70, 6.16, 1.97, 2.02, and 3.78 folds in Mo17 seeds/seedlings.

**Figure 6:**
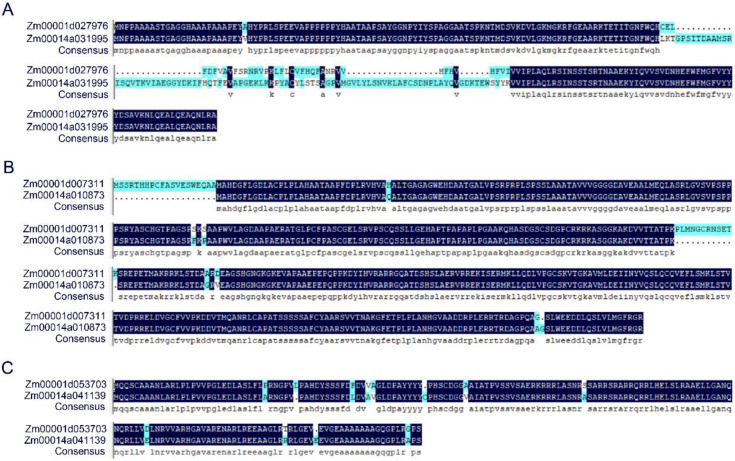
Sequence analysis of candidate genes between B73 and Mo17. Comparison of *Zm00001d027976* or *Zm00014a031996* (**A**), *Zm00001d007311* or *Zm00014a010873* (**B**), and *Zm00001d053703* or *Zm00014a041139* (**C**) amino sequence in B73 or Mo17 genome.

Low-temperature germination may not only be controlled by expression of candidate genes, which may be affected by the variation in coding sequence of candidate genes. The amino sequences of 39 QTL located genes, which were derived from B73 or Mo17 genomes, were used for alignment. There were only four genes that shared 100% similarity in B73 and Mo17 (Table S1). Insertion, deletion, and SNPs were distributed in amino sequence of remining genes. For example, *Zm00001d053703* (B73) had a large fragment insertion in amino sequence compared to *Zm00014a031996* (Mo17) (Figure 6A). *Zm00001d053703* (B73) had two fragment deletion compared to *Zm00014a041139* (Mo17) (Figure 6B). There were 11 SNPs that existed between the amino sequence of *Zm00001d053703* (B73) and *Zm00014a041139* (Mo17) (Figure 6C). All these variations in amino sequences may be responsible for different maize low-temperature tolerance.

## 3. Discussion

Early sowing will help maize reduce the effects of high temperature and drought on pollination and increase the yield of crop [20,21,22]. Maize production had been increased 17–33% by early sowing in Northeast China [23]. Low-temperature germination tolerance is necessary in early sowing of maize. Previously, the studies of cold tolerance were reported in many plants, such as rice, soybean, sorghum, and cucumber [24,25,26,27]. In rice, many QTLs related to low-temperature tolerance were located, and two major QTLs, *qLTG3-1* and *CTB4a*, were successfully applied to enhance low-temperature adaptability in breeding projects [5,28]. So far, QTLs related to low-temperature tolerance of maize were mainly focused on the seedling stage. Indexes such as chlorophyll fluorescence parameters, anthocyanin content tissue dry/fresh weight, leaf greenness, and leaf area were used in previous studies on low-temperature germination [11,29,30]. Here, plumule length, seedling length, root length, germination rate, germination index, vigor index, simple vigor index, and average germination days were used for QTLs in optimum- or low-temperature conditions (Table 3). 

In plants, natural populations and parental populations were widely used for association analysis related to low-temperature germination ability [4,6,9,16]. The natural population had higher recombinant efficiency and association accuracy, but this was limited by the population structure and local kinship. Parental population, such as DH population, had stable population structure, but it was affected by recombination efficiency. IBM Syn10 DH population used in this study was obtained by free pollination of F2 population of B73 × Mo17 for 10 generations, and then doubling haploid, which overcame the disadvantage of low recombination efficiency. In addition, 6618 SNP markers were used to construct a high-density genetic linkage map, and the average physical distance between the markers was 0.31 Mb (Figure 4). The average physical distances from the chromosome 1 to chromosome 10 were 0.27 Mb, 0.30 Mb, 0.32 Mb, 0.32 Mb, 0.28 Mb, 0.30 Mb, 0.31 Mb, 0.34 Mb, 0.34 Mb, and 0.40 Mb, respectively (Figure 4). All these greatly reduced the possibility of missing real QTLs.

The length of seedling, plumule, and mesocotyl were used as indexes of seed germination in abiotic stress [31,32]. In the low-temperature condition, the mesocotyl was too short to measure accurately. Root length (RL), stem length (SL), seed vigor index (SVI), average germination time, germination index, and plumule length were widely used for phenotype analysis during germination [33,34]. Therefore, the length of seedling and plumule were used as indexes of seed germination under the low-temperature condition (Figure 3). In our study, the correlation coefficient (r) between LTSL and LTPL was 0.982, while that between LTSL and LTRL, LTGR, LTGI, LTVI, and LTSVI was 0.675, 0.254, 0.405, 0.500, and 0.658, respectively (Table 2). The order of correlation coefficient was as follows: LTPL > LTRL > LTSVI > LTVI > LTGI > LTGR (Table 2). In further study, two overlapped regions, 18.575–18.875 Mb on chromosome 1 and 187.475–187.725 on chromosome 3 were located by QTLs related to LTPL and LTSL (Table 2). The region of 18.575–18.875 Mb on Chr. 1 explained 11.81% or 11.28% of LTPL or LTRL phenotypic variance, while the region of 187.475–187.725 Mb explained 3.73% or 5.56% of LTPL or LTRL phenotypic variance (Table 3). In a previous study, the traits of germination rate and germination index under low temperature were used for QTL analysis, and four regions of chromosomes 4, 5, 6, and 6 were located [9]. In this study, the QTL related to germination index, *qLTGI4-1*, was found on chromosome 4 under low-temperature, but no allele related to germination rate under low-temperature was found (Figure 4; Table 3). Average germination rate was an important index related to germination, but it is rarely used for QTL in previous study. In this study, LTAGD was negatively correlated with LTPL, LTSL, LTRL, LTGR, LTGI, LTVI, and LTSVI (Table 2). The order of correlation coefficient (r) was as follows: LTGI > LTSL > LTPL > LTVI > LTRL > LTSVI > LTGR (Table 2). *qLTAGD2-1*, which explained 6.99% of total phenotypic variance, had −0.64 of additive effect, suggesting the alleles from B73 background reduce average germination days and promote seed germination at low-temperature condition. In rice, plumule length and germination index were used as germination traits for QTL analysis under cold stress [33]. Here, three regions, *qLTPL1-1*, *qLTPL3-1*, and *qLTPL9-1*, were mapped to plumule length during germination under the low-temperature condition, while *qLTGI4-1* was associated with germination index (Table 3). In maize, seedling traits, including seedling root length, shoot length, and total length, were used for QTL mapping of low-temperature germination ability, and *mQTL1-1* was linked with seedling traits [35]. Here, *qLTSL1-1* and *qLTSL3-1* related to seedling length were identified (Table 3).

Different populations were used for association analysis of low-temperature germination ability in maize seedling stage and germination stage [1,15,16]. At maize seedling stage, S_221805316 related to seed vigor under cold treatment, which was mapped by QTL using B73 × P39 RIL population [12], was overlapped with *qLTAGD2-1* in our study, the region at 220.475~221 Mb on chromosome 2 associated with average germination days under the low-temperature condition (Table 3). At germination stage, 30 single-nucleotide polymorphisms (SNPs) related to low-temperature tolerance were mapped by GWAS [16]. Among them, SYN21841, located at 219,117,623 bp of chromosome 2, related to relative radicle volume of maize germinated seeds under low-temperature condition, was also in a nearby region of *qLTAGD2-1* (Table 3). In the field at early sowing dates, the S1_18888944, at the position of S1_18.89 Mb on chromosome 1, was associated with Fv/Fm using MAGIC population [10], which was near the region of 18.575–18.875 Mb, the overlapped region of *qLTPL1-1* and *qLTSL1-1* in our study (Table 3). In addition, chr03.1873.5, chr04.2343.5, and chr09.1427.5 (Table 3), which were associated with low-temperature germination ability in our study, were not detected in previous studies. This indicates that the specific QTL regions could be excavated by different populations and markers.

The effects of candidate genes on low-temperature germination tolerance of B73 and Mo17 mainly focus on two aspects: the expression levels of candidate genes and the variation of amino sequence. Here, eight QTL located genes, which exhibited expression difference in B73 and Mo17 seeds/seedlings, were found by joint analysis of RNA-Seq and QTL located genes (Figure 5). Among them, *Zm00001d043166*, which was located at the *qLTPL3-1* and *qLTSL3-1* overlapped region, was encoded UDP- glycosyltransferase 87A1 (Table S1). In Arabidopsis, UDP-glycosyltransferases UGT79B2 and UGT79B3 could improve cold stress tolerance by accumulated anthocyanin [36]. *Zm00001d043166* may promote elongation of plumule and seedling under low temperature. *Zm00001d007315*, which was encoded GID2, was located at *qLTAGD2-1* region (Table S1). In Arabidopsis, CPK3-phosphorylated RhoGDI1 could regulate the development of seedlings [37]. Development of seedlings was negatively correlated with germination days; *Zm00001d007315* may shorten the germination days by promoting seedling development. *Zm00001d027974* was located at the overlapped region of *qLTPL1-1* and *qLTSL1-1* and encoded an ABC transporter (Table S1). *AtABCB21* could affect development of Arabidopsis by adjusted Auxin concentration [38]. 

Additionally, the amino sequences of QTL located genes were used for comparison. Amino sequence of thirty genes was varied between B73 and Mo17 (Table S1). The differentiation of low-temperature germination tolerance between B73 and Mo17 may be caused by variation of amino sequence. *Zm00001d027976* was located at the overlapped region of *qLTPL1-1* and *qLTSL1-1* and encoded GLABRA2 (Table S1). Arabidopsis GLABRA2, which regulated the biosynthesis of anthocyanin, played important roles in abiotic stress [39]. In poncirus trifoliata, cold sensitivity was elevated in *PtrbHLH* RNAi lines, in which POD activity was decreased [40]. In fruit colouration, MdbHLH3 could regulate low-temperature-induced anthocyanin accumulation [41]. In maize, bHLH55 positively affected salt tolerance by regulating the biosynthesis of AsA [42]. *Zm00001d007311,* which encoded bHLH transcription factor, was included in the region of *qLTAGD2-1*(Table S1). In postharvest kiwifruit, *AchnABF1*, a bZIP gene, negatively regulated freezing stress [43]. Freezing tolerance was decreased in wheat *TabZIP6* overexpressing Arabidopsis, in which the expression of CBFs was downregulated [44]. *Zm00001d053703*, which encoded a bZIP transcription factor, was located at region of *qLTGI4-1* (Table S1). Taken altogether, we believed that the expression difference of *Zm00001d043166*, *Zm00001d007315*, *Zm00001d027974* and the amino sequence difference of *Zm00001d027976*, *Zm00001d007311*, *Zm00001d053703* between B73 and Mo17 may be responsible for the difference of low-temperature germination ability of B73 and Mo17.

In summary, five QTLs clusters, related plumule length, seedling length, germination index, and average germination days under low-temperature condition were mapped by IBM Syn 10 population. In total, 39 genes were extracted from these five regions. Joint analysis with RNA-Seq at germinated seeds/seedling under low-temperature, three genes upregulated in B73 and five genes upregulated in Mo17 were excavated from QTL-located genes. Additionally, the variations, such as insertion, deletion, and SNPs, were presented in an amino sequence of B73 and Mo17. The expression levels and amino sequence variation of candidate genes may be responsible for the difference of low-temperature germination tolerance between B73 and Mo17. The study will provide a theoretical basis for marker-assisted breeding and lay a foundation for further revealing molecular mechanism of low-temperature germination tolerance in maize.

## 4. Materials and Methods

### 4.1. Plant Material

A population of 176 lines, the intermated B73 × Mo17 (IBM) Syn10 DH population, was used for QTL analysis of low-temperature germination ability. The IBM Syn10 DH population was obtained by free pollination of F2 population of B73 × Mo17 for 10 generations and then doubling haploid [32].

### 4.2. Germination Conditions and Measurement Indexes

Germination experiments were performed in a growth chamber at 10 °C. Three repetitions were undertaken for each treatment during germination, with 30 seeds per repetition. A germination experiment was conducted in internal paper, and the method details are as follows: firstly, maize seeds were laid on sterilized paper (45.0 cm × 30.5 cm); secondly, the sterilized paper on which the seeds were put was rolled and fixed; thirdly, this was put into plastic bags, and enough sterile water was added to ensure seed germination; finally, the rolling paper was erected in the germination basin, in which 2 cm depth water was filled, at 10 °C (8 h/16 h, light/dark) for 21 days, while the seeds germinated at 25 °C (8 h/16 h, light/dark) were used as a control. The number of germinated seeds was counted every day from the second day. Root length (RL), plumule length (PL), and seedling length (SL) were measured after 7 days in optimum-temperature condition or 21 days in low-temperature condition, and then germination rate (GR), germination index (GI), vigor index (VI), simple vigor index (SVI), and average germination days (AGD) were calculated as in a previous paper [45].

The average value, maximum value, minimum value, standard deviation (s), and significance were analyzed by software SPSS 21.0. CV (the coefficients of variation, %) for each trait were calculated as follows: CV = s/$\bar{x}$, where s is the standard deviation [15].

### 4.3. QTL Analysis and Candidate Gene Mining

A bin map with 6618 recombination bins were constructed in IBM Syn 10 DH populations in a previous study [19]. IBM genotype data used in this research were referred as previous studies [19]. QTL mapping analysis was carried out for each trait related to germination of IBM Syn 10 DH population. Using QTL lciMapping software, and following an inclusive composite interval mapping (ICIM) pattern, we constructed a high-density genetic linkage map containing 6618 bin markers for QTL analysis [19]. LOD (logarithm of ODS) = 2.5, as the threshold value was set for every trait to map QTLs and determine the mode of action of QTLs. The QTL was named according to the rule of McCouch’ paper [46]. The physical coordinates of QTL were referred to B73_RefGen_v2. The conversion of different genomic version was conducted on the website (https://ensembl.gramene.org/Zea_mays/Tools/AssemblyConverter, accessed on 18 May 2021). In total, 39 genes that have annotations were extracted from five overlapping intervals based on B73_RefGen_v4 in Maize Genetics and Genomics Database (https://www.maizegdb.org/gene_center/gene#list, accessed on 19 May 2021) (Table S1).

### 4.4. RNA-Seq Analysis and Candidate Gene Selection

For RNA-Seq analysis of maize seeds/seedling, maize B73 and Mo17 were geminated in 10 °C for 10 d. The whole seeds/seedlings were collected and sent to Biomarker Technologies Co, LTD, Beijing, China. for RNA extraction and sequencing. There are three replicates for each treatment, and each replicate includes five seeds/seedlings. The differentially expressed genes, which had a change fold > 1.5 or < 0.67 in B73 compared to Mo17, between B73 and Mo17 were provided in Table S2.

Comparison of the genes included in the QTL region and the genes upregulated in B73 or Mo17 seeds/seedling under low temperature was performed using online software (http://www.interactivenn.net/, accessed on 19 May 2021) [47].

For sequence analysis, the amino sequences of candidate genes in B73 (RefGen_v4; CSHL) or Mo17 (CAU gene models; Zm00014a) were downloaded from Maize Genetics and Genomics Database (http://www.maizegdb.org, accessed on 19 May 2021). Sequence comparisons were conducted by DNAMAN software. The expressions of candidate genes in B73 or Mo17 were extracted from Table S2, and statistical analysis was conducted by Student’s *t*-test using IBM SPSS Statistics 19.0 software (International Business Machines Corporation, Armonk, NY, USA).

## Figures and Tables

**Table 1:** Germination-related traits of Syn10 population under optimum or low temperature.

Traits ^a^	Parent ^b^		Syn10 Population			
	Mo17	B73	Range	Mean	S ^c^	CV ^d^
OTPL *	16.9	18.5	7.9–28.0	16.9	3.4	20
OTSL **	8	13.6	3.3–21.8	11.2	3.5	31.5
OTRL *	9.2	15	4.0–23.0	12.6	3.6	28.7
OTGR	93.3	100	85.0–100.0	96.6	3.8	4
OTGI	3	3.1	2.3–5.9	3.1	0.6	18.4
OTVI *	54.5	59.4	24.8–85.4	51.8	10.1	19.5
OTSVI *	1695.4	1899.7	981.7–2600.0	1668.5	261.6	15.7
OTAGD	3.2	3.3	2.1–4.5	3.3	0.4	12.2
LTPL ***	0.8	2.2	0.3–5.5	1.5	1.0	67.8
LTSL **	1.3	2.7	0.5–6.3	2.1	1.1	51.7
LTRL ***	0.6	6.8	0.1–9.1	3.3	1.7	52.7
LTGR *	83.3	96.7	20.0–100.0	84.8	15.6	18.4
LTGI **	0.5	2.6	0.1–2.3	0.7	0.3	38.3
LTVI ***	0.4	18.1	0.1–21.8	2.6	2.4	94.2
LTSVI **	57.7	661.7	19.0–853.7	294.6	170.0	57.7
LTAGD **	16	7.4	8.0–19.9	13.4	2.3	17.1

^a^ OTPL, OTSL, OTRL, OTGR, OTGI, OTVI, OTSVI, and OTAGD represent plumule length, seedling length, root length, germination rate, germination index, vigour index, simple vigour index, and average germination days under optimum temperature, respectively; LTPL, LTSL, LTRL, LTGR, LTGI, LTVI, LTSVI, and LTAGD represent plumule length, seedling length, root length, germination rate, germination index, vigour index, simple vigour index, and average germination days under low temperature, respectively; ^b^ Asterisk represents significant difference between Mo17 and B73 for each trait. * *p* < 0.05, ** *p* < 0.01, *** *p* < 0.001 (Student’s *t* test); ^c^ S represents standard deviation; ^d^ CV represents coefficient of variation.

**Table 2:** Correlation coefficients (r) between plumule length, seedling length, root length, germination rate, germination index, vigor index, simple vigor index, and average germination days of IBM Syn10 population under optimum or low temperature.

Traits	OTPL (cm)	OTSL (cm)	OTRL (cm)	OTGR (%)	OTGI	OTVI	OTSVI	OTAGD (d)	LTPL (cm)	LTSL (cm)	LTRL (cm)	GR (%)	LTGI	LTVI	LTSVI	LTAGD (d)
OTPL (cm)	1.000															
OTSL (cm)	0.627 **	1.000														
OTRL (cm)	0.626 **	0.997 **	1.000													
OTGR (%)	0.177 *	0.165 *	0.172 *	1.000												
OTGI	0.101	0.184 *	0.187 *	0.591 **	1.000											
OTVI	0.553 **	0.452 **	0.448 **	0.522 **	0.782 **	1.000										
OTSVI	0.672 **	0.468 **	0.466 **	0.693 **	0.424 **	0.811 **	1.000									
OTAGD (d)	−0.001	−0.098	−0.097	0.065	−0.716 **	−0.510 **	0.025	1.000								
LTPL (cm)	0.053	0.078	0.079	−0.063	0.227 **	0.139	−0.049	−0.310 **	1.000							
LTSL (cm)	0.064	0.076	0.077	−0.056	0.238 **	0.152 *	−0.036	−0.326 **	0.982 **	1.000						
LTRL (cm)	0.255 **	0.347 **	0.354 **	0.003	0.132	0.169 *	0.102	−0.161 *	0.670 **	0.675 **	1.000					
LTGR (%)	0.170 *	0.264 **	0.269 **	0.115	0.181 *	0.219 **	0.176 *	−0.176 *	0.204 **	0.254 **	0.375 **	1.000				
LTGI	0.043	0.128	0.133	0.177 *	0.265 **	0.166 *	0.072	−0.198 **	0.380 **	0.405 **	0.574 **	0.537 **	1.000			
LTVI	0.123	0.211 **	0.216 **	0.080	0.151 *	0.114	0.055	−0.125	0.498 **	0.500 **	0.817 **	0.346 **	0.875 **	1.000		
LTSVI	0.265 **	0.367 **	0.374 **	0.013	0.132	0.183 *	0.123	−0.163 *	0.646 **	0.658 **	0.971 **	0.547 **	0.634 **	0.823 **	1.000	
LTAGD (d)	−0.037	−0.094	−0.095	−0.007	−0.295 **	−0.219 **	−0.019	0.368 **	−0.605 **	−0.642 **	−0.503 **	−0.200 **	−0.692 **	−0.597 **	−0.486 **	1.000

OTPL, OTSL, OTRL, OTGR, OTGI, OTVI, OTSVI, and OTAGD represent plumule length, seedling length, root length, germination rate, germination index, vigor index, simple vigor index, average germination days under optimum temperature, respectively; LTPL, LTSL, LTRL, LTGR, LTGI, LTVI, LTSVI, and LTAGD represent plumule length, seedling length, root length, germination rate, germination index, vigor index, simple vigor index, and average germination days under low temperature, respectively. Asterisk represents significant difference. * *p* < 0.05, ** *p* < 0.01 (Student’s *t* test).

**Table 3:** Genome-wide QTL identification under optimum and low temperature in IBM Syn10 population.

QTL Symbol ^a^	Chr	Position (cm)	Left Marker	Right Marker	LOD ^b^	Additive Effect ^c^	PVE ^d^ (%)	2-LOD Confidence Interval			
								GC ^e^ Start (cM)	GC End (cM)	PC ^f^ Start (Mb)	PC End (Mb)
*qOTPL8-1*	8	53	chr08.243.5	chr08.244.5	2.68	−0.97	7.06	52.5	53.5	24.275	24.4
*qOTGI3-1*	3	143	chr03.2044.5	chr03.2045.5	2.79	0.14	6.24	142.5	143.5	204.4	204.5
*qOTVI4-1*	4	168	chr04.2370.5	chr04.2371.5	3.06	−4.08	7.09	167.5	169.5	237	237.1
*qOTVI6-1*	6	132	chr06.1642.5	chr06.1643.5	4.35	−5.17	8.32	131.5	132.5	164.2	164.3
*qOTVI8-1*	8	103	chr08.1655.5	chr08.1656.5	2.51	3.68	5.51	102.5	103.5	165.5	165.6
*qOTVI9-1*	9	101	chr09.1427.5	chr09.1428.5	2.53	3.34	4.81	100.5	101.5	142.7	142.8
*qLTPL1-1*	1	50	chr01.187.5	chr01.190	7.02	0.37	11.81	49.5	50.5	18.575	18.875
*qLTPL3-1*	3	126	chr03.1873.5	chr03.1876	3.67	0.25	5.56	125.5	126.5	187.475	187.725
*qLTPL9-1*	9	101	chr09.1427.5	chr09.1428.5	4.26	0.28	6.82	100.5	101.5	142.7	142.8
*qLTSL1-1*	1	50	chr01.187.5	chr01.190	8.17	0.47	11.28	49.5	50.5	18.575	18.875
*qLTSL3-1*	3	126	chr03.1873.5	chr03.1876	3.02	0.27	3.73	125.5	126.5	187.475	187.725
*qLTGI4-1*	4	156	chr04.2343.5	chr04.2344.5	2.92	−0.07	6.82	155.5	156.5	234.3	234.4
*qLTAGD2-1*	2	172	chr02.2206.5	chr02.2207.5	3.85	−0.64	6.99	171.5	172.5	220.475	221

^a^ OTPL, OTSL, OTRL, OTGR, OTGI, OTVI, OTSVI, and OTAGD represent plumule length, seedling length, root length, germination rate, germination index, vigor index, simple vigor index, and average germination days under optimum temperature, respectively; LTPL, LTSL, LTRL, LTGR, LTGI, LTVI, LTSVI, and LTAGD represent plumule length, seedling length, root length, germination rate, germination index, vigor index, simple vigor index, and average germination days under low temperature, respectively. ^b^ LOD: Log10-likelihood value. ^c^ Additive effect: the positive value means that the allele from B73 is positive contributor. ^d^ PVE represents phenotypic variance explained. ^e^ GC represents genetic coordinates. ^f^ PC represents physical coordinates refer to B73_RefGen_v2.

## Data Availability

Not applicable.

## Supplementary Materials

The following supporting information can be downloaded at: https://www.mdpi.com/article/10.3390/plants11020214/s1. Table S1: Candidate genes included in QTL region controlling low-temperature germination. (Please refer to Excel file). Table S2: Genes upregulated in B73 or Mo17 seeds/seedlings under low-temperature conditions (Please refer to Excel file).
